# The psychological restorative effects of green exercise: a moderated mediation model of nature relatedness and exercise behavior

**DOI:** 10.3389/fspor.2026.1887724

**Published:** 2026-07-09

**Authors:** Tzu-Chun Huang, Ching Li, Ming-Wei Huang

**Affiliations:** 1 Graduate Institute of Sport, Leisure and Hospitality Management, National Taiwan Normal University, Taipei, Taiwan; 2 Department of Tourism and Leisure Management, Hsing Wu University, New Taipei City, Taiwan

**Keywords:** cognitive resource allocation, conditioned restoration theory, outdoor physical activity, perceived environmental restorativeness, PLS-SEM

## Abstract

Green exercise yields synergistic physical and mental health benefits; however, the psychological transformative mechanisms underlying these effects warrant further investigation. Grounded in Conditioned Restoration Theory (CRT), the main purpose of this study was to construct and validate a theoretical model of the psychological restoration pathways within green exercise contexts. Using a stratified sampling method, data were collected from 1,098 participants in Taiwan and analyzed via Partial Least Squares Structural Equation Modeling (PLS-SEM). The results revealed that Perceived Naturalness (PN) significantly enhanced Perceived Environmental Restorativeness (PER) both directly and indirectly through Nature Relatedness (NR). Furthermore, exercise behavior significantly and negatively moderated the direct pathway from PN to PER. This attenuating interaction effect suggests that vigorous or prolonged physical exertion may induce cognitive resource competition, thereby diminishing the restorative benefits derived from the natural environment. These findings not only broaden the theoretical framework for understanding the psychological restoration mechanisms of green exercise but also offer critical practical implications for public health and urban planning.

## 1 Introduction

Green exercise — defined as physical activity undertaken in natural environments — yields synergistic physical and psychological restorative benefits (1, 2). The psychological restoration pathways of green exercise have been grounded in two classic theories: The Stress Reduction Theory (SRT), derived from evolutionary psychology (3), and the Attention Restoration Theory (ART), which emphasizes cognitive processing (4). While evolutionary theories explain nature-induced physiological recovery, cognitive frameworks address attention restoration via soft fascination. Given that cognitive fatigue triggers physiological stress and attention relief fosters positive affect, SRT and ART are highly likely to be complementary and concurrent (5). Consequently, Egner, Sütterlin (6) proposed the Conditioned Restoration Theory (CRT) as an integrative framework, positing that the benefits of nature exposure are the dual product of evolutionary instincts and conditioned learning.

According to CRT, an individual’s past recreational experiences in nature transform environmental cues into conditioned stimuli that induce relaxation (7). This reveals that implicit attitudes, such as Nature Relatedness (NR) or environmental preference, may serve as critical boundary conditions for psychological restorative mechanisms (8, 9). However, most studies in the field of green exercise have focused strictly on comparing different environmental conditions (10–13) or specific exercise modalities (14–16). While a recent study has investigated how inter-individual differences, such as nature connectedness and childhood exposure, modulate immediate affective responses to different environments (17), these findings primarily highlight how such traits differentially affect responses to urban versus natural scenes. Consequently, the role of individuals’ implicit attitudes within dynamic human-environment interactions remains largely underexplored. Furthermore, although preliminary empirical work by Wood and Smyth (18) explicitly indicated that an individual’s subjective connection to nature can predict subsequent restorative states, the failure to account for specific exercise behaviors limits a comprehensive understanding of the green exercise process. From the perspective of cognitive resource allocation, high-intensity or prolonged exercise shifts an individual’s directed attention toward internal physiological signals, such as regulating breathing and enduring fatigue. This shift creates resource competition with the processing of external natural environmental information, thereby interfering with the restorative effects (19).

To address these research gaps, the main purpose of this study was to construct and validate an integrated moderated mediation model to clarify the psychological restoration pathways in green exercise contexts, grounded in CRT and cognitive resource allocation perspectives. Specifically, this study aimed to address the following questions:

1. Did an individual’s Perceived Naturalness (PN) of the environment significantly enhance their Perceived Environmental Restorativeness (PER) during green exercise?
2. Did NR mediate the relationship between PN and PER?
3. Did exercise behavior moderate the pathway from PN to PER?

**Figure 1:**
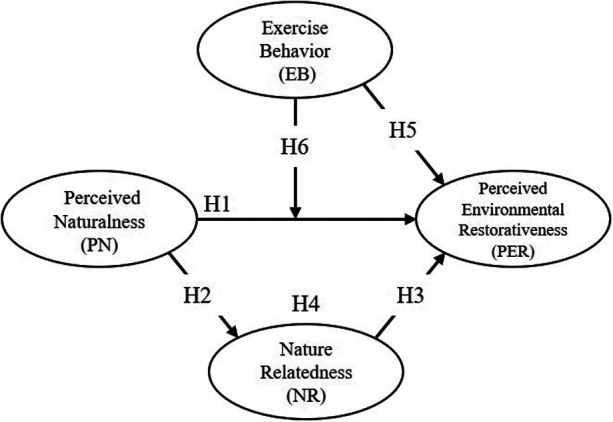
The proposed conceptual model and research hypotheses.

Drawing upon the aforementioned theoretical frameworks, the following hypotheses were formulated, and the overall conceptual model is depicted in Figure 1.

Rooted in SRT and ART, highly natural environments not only directly alleviate fatigue but also evoke emotional resonance and a connection with nature. Therefore:

H1: PN would positively affect PER.
H2: PN would positively affect NR.
H3: NR would positively affect PER.

Furthermore, CRT suggests that environmental stimuli must be translated into actual restorative experiences through an individual’s implicit attitudes. Hence:

H4: NR would significantly mediate the relationship between PN and PER.

Finally, while green exercise inherently provides physical and mental benefits, an excessive exercise load may induce cognitive resource competition, thereby attenuating the primary restorative effect derived purely from environmental naturalness. Thus:

H5: Exercise behavior (EB) would positively affect PER.
H6: EB would significantly moderate the positive effect of PN on PER.

## 2 Method

### 2.1 Data collection

This study employed a quantitative survey design. The target population comprised healthy individuals aged 13 years and older residing across the 13 administrative districts of Taoyuan City, Taiwan. To ensure sample representativeness, a proportional stratified sampling method was utilized, distributing the surveys in accordance with the actual population distribution of each district. Data collection occurred between February and April 2025. All study procedures were reviewed and approved by the Research Ethics Committee of National Taiwan Normal University (Protocol Number: 202503HS033) prior to data collection.

A total of 1,098 valid responses were obtained. The sample comprised 571 males (52.0%) and 527 females (48.0%). To justify the adequacy of the sample size for Partial Least Squares Structural Equation Modeling (PLS-SEM), an *a priori* power analysis was conducted. Assuming a medium effect size, a significance level of *α* = .05, and a statistical power of 0.8, the final sample size substantially exceeded the required minimum threshold, ensuring robust statistical power for the subsequent analyses.

### 2.2 Measures

All measurement instruments were administered in Traditional Chinese. The NR scale, originally developed in English, underwent a rigorous translation and back-translation procedure by independent bilingual researchers. Subsequently, the complete questionnaire was reviewed and refined by an expert committee comprising six scholars and industry practitioners (detailed profiles are provided in Table 2 of the Supplementary Material) to ensure optimal face validity, clarity, and cultural appropriateness for the target population.

#### 2.2.1 Perceived naturalness

The perceived naturalness of the exercise environment was assessed using a single item adapted from the environmental setting classification of the Recreation Opportunity Spectrum (ROS) (20, 21). Participants were asked to select the description that best characterized their usual exercise location. Responses were recorded on a 5-point rating scale spanning five distinct environmental settings: (I) built environments without green natural elements (e.g., enclosed sports facilities); (II) built environments with green natural elements (e.g., plantings, natural landscaping); (III) urban parks and green spaces (e.g., large-scale sports parks); (IV) facilities surrounded by natural elements (e.g., hiking trails, managed beaches); and (V) primitive natural environments (e.g., wilderness, pathless deep mountains). Higher scores indicate a greater degree of perceived naturalness in the exercise environment. By embedding these concrete examples within each description, respondents could readily retrieve and connect with their experiences, thereby facilitating an intuitive environmental self-localization.

#### 2.2.2 Nature relatedness

Individuals’ implicit connection to nature was measured using the short-form Nature Relatedness Scale (NR-6) developed by Nisbet and Zelenski (22). This 6-item instrument asks participants to rate their agreement with statements regarding their affective and cognitive relationship with the natural world (e.g., “My ideal vacation spot would be a remote, wilderness area”). Responses were provided on a 5-point Likert scale ranging from 1 *(strongly disagree)* to 5 *(strongly agree)*. Item scores were averaged, with higher values reflecting a stronger sense of nature relatedness.

#### 2.2.3 Perceived environmental restorativeness

The perceived restorative quality of the exercise environment was evaluated using the 15-item Perceived Restoration Scale validated by Huang and Li (23), which is grounded in Kaplan and Kaplan’s (4) ART. The scale captures four dimensions of restorativeness: being away, extent, fascination, and compatibility. Participants rated their agreement with statements regarding their current exercise environment (e.g., “In this place, I can feel a different atmosphere from my regular environment”) using a 5-point Likert scale ranging from 1 *(strongly disagree)* to 5 *(strongly agree)*. A mean score was calculated, with higher scores representing greater perceived environmental restorativeness.

#### 2.2.4 Exercise behavior

Exercise behavior was operationalized through two dimensions — exercise duration and perceived intensity — adapted from the Taiwan National Sports Survey (24). Exercise duration was measured as a continuous variable by asking participants to report their average exercise time per session (“How many minutes do you exercise on average per session?”). Exercise intensity (Rating of Perceived Exertion, RPE) was assessed based on physiological responses (sweating and panting) during their most frequent exercise. Responses were coded on a 4-point ordinal scale ranging from 1 (light intensity; neither sweating nor panting) to 4 (vigorous intensity; both sweating and panting).

To capture the overall physical exertion for the PLS-SEM moderation analysis, a single composite index representing the total “exercise behavior” was calculated by multiplying the exercise duration by the perceived intensity score for each participant.

### 2.3 Data analysis

Data were analyzed and hypotheses were tested using Partial Least Squares Structural Equation Modeling (PLS-SEM) via SmartPLS 4 (25). PLS-SEM was selected over covariance-based SEM for two primary reasons. First, it is a robust, nonparametric method well-suited for the non-normally distributed data frequently encountered in social science research. Second, the dual objective of this study—encompassing both theoretical explanation and out-of-sample prediction—aligns strongly with the causal-predictive nature of PLS-SEM (26, 27).

Following contemporary guidelines, a two-step evaluation procedure was adopted (28). In the first phase, the measurement model was evaluated according to the nature of the constructs (reflective vs. formative). For the reflective constructs, internal consistency reliability was assessed using Cronbach’s *α* and composite reliability (CR), with values ≧ 0.7 indicating satisfactory reliability. Convergent validity was established by ensuring the average variance extracted (AVE) was ≧ 0.5 and outer loadings were ideally > 0.7. Discriminant validity was rigorously evaluated using the Heterotrait-Monotrait (HTMT) ratio, adhering to a conservative threshold of < 0.85. For the formative construct, indicator collinearity was first examined using the Variance Inflation Factor (VIF), applying a strict threshold of < 3. Subsequently, the indicator contributions were assessed by examining the significance of outer weights (relative contribution). If an indicator’s weight was non-significant, its outer loading was evaluated; indicators were retained if their outer loading was ≧ 0.5, indicating a significant absolute contribution to the construct (28).

In the second phase, the structural model was evaluated. Potential collinearity among predictor constructs was checked using the inner VIF, ensuring values remained below 3 to guarantee stable path estimations (26). To assess the significance of direct, specific indirect (mediation), and moderation effects, a bootstrapping procedure with 10,000 subsamples was executed to generate path coefficients, t-values, *p*-values, and 95% confidence intervals (28). The model’s explanatory power was evaluated using the coefficient of determination (*R^2^*). Furthermore, the substantive impact of each path was assessed via the effect size (*f^2^*), applying Cohen’s criteria (.02,.15,.35 for small, medium, and large effects) (29). For the moderation analysis, adjusted standards (.005,.010,.025) were utilized, acknowledging that interaction terms typically yield substantially smaller effect sizes in applied social sciences (30).

## 3 Results

### 3.1 Measurement model assessment

The model comprises three reflective constructs (i.e., NR, PER, and PN) and one formative construct (i.e., EB). The outer loadings, weights, and variance inflation factor (VIF) values for all items are detailed in Table 1.

**Table 1:** Measurement model evaluation.

Construct	Type	Indicator	Loading/Weight	VIF
Nature Relatedness (NR)	Reflective	NR-1	.633	1.399
		NR-2	.672	1.505
		NR-3	.726	1.624
		NR-4	.863	2.773
		NR-5	.865	2.812
		NR-6	.821	2.197
Perceived Environmental Restorativeness (PER)	Reflective	PER-1	.667	1.852
		PER-2	.682	2.025
		PER-3	.629	1.696
		PER-4	.775	2.257
		PER-5	.631	1.594
		PER-6	.741	2.057
		PER-7	.697	1.799
		PER-8	.756	2.064
		PER-9	.724	2.314
		PER-10	.774	2.743
		PER-11	.804	2.916
		PER-12	.766	2.373
		PER-13	.785	2.641
		PER-14	.766	2.346
		PER-15	.680	1.929
Perceived Naturalness (PN)	Reflective	PN	1.000	–
Exercise Behavior (EB)	Formative	RPE	.833 (w)	1.027
		DUR	.435 (w)	1.027

Outer loadings are reported for reflective constructs; outer weights, denoted by (w), are reported for formative constructs. All Variance Inflation Factor (VIF) values are below the strict threshold of 3.0, indicating the absence of indicator-level collinearity issues.

The reliability and validity of the reflective measurement models were assessed first. As shown in Table 2, the Cronbach’s alpha values for NR and PER were.859 and.936, respectively, and their composite reliability (CR) values were.895 and.944. All values exceeded the recommended threshold of.70, indicating excellent internal consistency reliability. Regarding convergent validity, the average variance extracted (AVE) for NR and PER were.591 and.529, respectively, both surpassing the.50 criterion. At the indicator level, most outer loadings exceeded the ideal threshold of.708. Although a few indicators (e.g., NR-1, PER-3) exhibited loadings between.629 and.697, they were retained to preserve content validity, as the overall CR and AVE for their respective constructs met the required standards (26). Additionally, all reflective indicators demonstrated VIF values below 3.0, confirming the absence of multicollinearity issues.

**Table 2:** Reliability, convergent validity, and discriminant validity (HTMT).

Constructs	Cronbach’s *α*	CR	AVE	HTMT			
				NR	PER	PN	EB x PN
PN	–	–	–				
NR	.859	.895	.591	.606			
PER	.936	.944	.529	.161	.179		
EB x PN	–	–	–	.021	.081	.089	

PN, perceived naturalness; NR, nature relatedness; PER, perceived environmental restorativeness; EB, exercise behavior. PN is measured as a single-item construct; thus, Cronbach’s α, CR, and AVE are not applicable.

Subsequently, the reliability and validity of the formative construct (EB) were evaluated by examining indicator collinearity and their absolute and relative contributions. The VIF values for both exercise intensity (RPE) and exercise duration (DUR) were 1.027, well below the 3.0 threshold, indicating no collinearity concerns. Regarding relative contribution, the outer weight for RPE was.833 and statistically significant. Although the outer weight for DUR (.435) was non-significant, its outer loading was.569. According to decision rules (28), formative indicators with non-significant weights but outer loadings above.50 should be retained due to their significant absolute contribution to the construct. Thus, DUR was retained to maintain the theoretical integrity of the EB construct.

Finally, discriminant validity was evaluated using the heterotrait-monotrait (HTMT) ratio of correlations. As presented in Table 2, all HTMT values between constructs ranged from.021 to.606. These values are strictly below the conservative threshold of.85, thereby establishing robust discriminant validity and high empirical distinctiveness among all constructs in the model.

### 3.2 Structural model assessment

**Figure 2:**
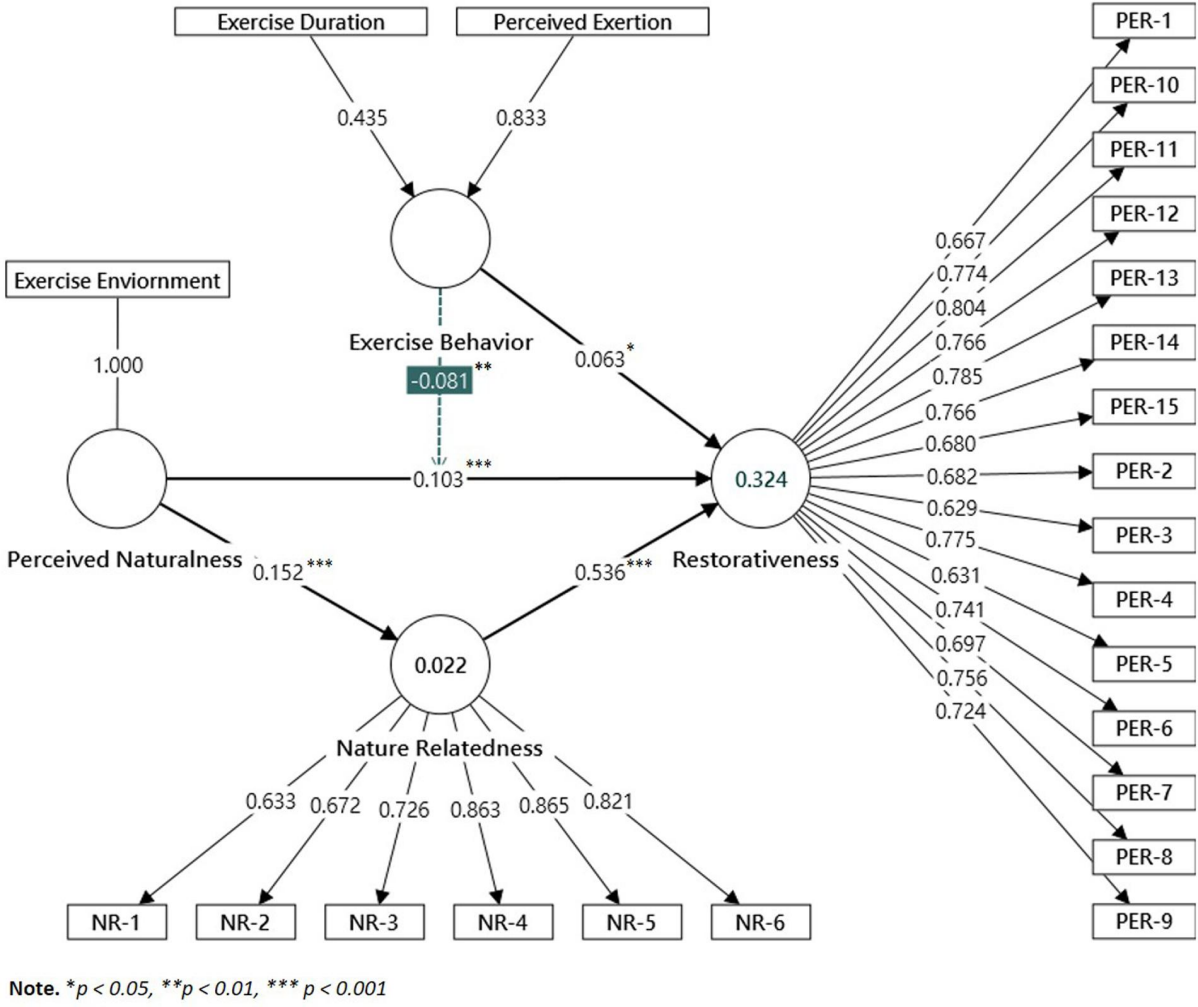
The psychological restoration pathways in green exercise.

After confirming the reliability and validity of the measurement model, the hypothesized structural model was assessed. The results, as shown in Figure 2, indicate that the hypothesized pathways were largely supported, with detailed statistics provided in Supplementary Material.

First, the inner model’s collinearity was examined. Inner variance inflation factor (VIF) values for all predictor constructs ranged from 1 to 1.074, falling strictly below the recommended < 3 (26). This confirms the absence of multicollinearity, ensuring high stability of the structural model estimates.

Subsequently, a bootstrapping procedure with 10,000 subsamples was executed to evaluate the specific pathways (28). The analysis revealed that PN had a significant positive direct effect on PER (*β* = .103, *p* < .001). Concurrently, PN exerted a significant indirect effect on PER through NR (*β* = .081, *p* < .001). Because both the direct and indirect effects were positive and statistically significant, NR acts as a complementary partial mediator in the model. The variance accounted for was 44.02%, indicating that this indirect pathway contributes substantially to the total effect. The structural model revealed that EB exerted a small but statistically significant positive direct effect on PER (*β* = .063, *p* < .05). Furthermore, the moderation analysis demonstrated that EB significantly and negatively moderated the pathway from PN to PER (*β* = -.081, *p* = .004). This finding implies that as the intensity or duration of EB increases, the primary restorative effect of PN gradually diminishes, exhibiting an attenuating interaction effect.

Regarding the model’s explanatory power, the coefficient of determination (*R*^2^) for the core endogenous construct, PER, was.327 (adjusted *R*^2^ = .324), representing a moderate level of explanatory power (26). In terms of the substantive contribution of individual paths, the pathway from NR to PER demonstrated a large effect size (*f^2^* = .415), confirming that NR is the most critical driver of perceived restorativeness. The pathway from PN to NR exhibited a small effect size (*f^2^* = .024). Notably, although the effect sizes for the direct paths from PN and EB to PER fell below conventional threshold criteria (26, 28), their corresponding path coefficients achieved high statistical significance, indicating meaningful predictive relevance within the model. Finally, the moderation effect size for the interaction term (EB X PN)(*f^2^* = .009) was not only larger than the direct effect of EB on PER but also approached a moderate level of practical significance based on the adjusted criteria for moderation in the applied social sciences (30). This demonstrates that EB critically influences the psychological conversion process from PN to PER.

To address potential common method bias (CMB) inherent in our single-session, self-reported survey design, we conducted the Full Collinearity VIF Approach recommended by Kock (31). As shown in Supplementary Table S3, the inner VIF values for all latent constructs (EB, NR, PER, and PN) range from 1.053 to 1.446. Since all values are well below the conservative threshold of 3.3, severe CMB can be confidently ruled out.

## 4 Discussion

### 4.1 The mediating role of nature relatedness: validating conditioned restoration theory

This study empirically tested a moderated mediation model integrating CRT and a cognitive resource allocation perspective to elucidate psychological restoration pathways in green exercise. Validating the core proposition of CRT, the primary finding reveals that an individual’s implicit NR serves as the critical psychological mechanism for generating the synergistic restorative benefits of green exercise. Specifically, while PN directly enhances PER (*β* = .103) in accordance with the fundamental assumptions of SRT (3) and ART (4), it is the activation of this pre-existing emotional connection to nature that fundamentally translates external natural stimuli into profound subjective recovery. This transformation is strongly supported by the robust indirect effect (*β* = .081), where NR acts as a complementary partial mediator accounting for a substantial 44.02% of the total effect (VAF) and exerting a large substantive effect on PER (*f^2^* = .415). This conversion mechanism conceptually extends prior empirical works, which suggest that individual traits inherently shape nature experiences (17, 18).

### 4.2 The moderating effect of exercise behavior: a cognitive resource competition perspective

The structural model results reveal a critical distinction between the direct and interactive effects of exercise behavior. The direct path from exercise behavior to perceived environmental restorativeness is minimal (*β* = .063, *f^2^* = .006). While statistically significant, this relationship possesses an extremely weak effect size, indicating that its significance is probably a consequence of the large sample size. Practically, although exercise behavior could potentially influence perceived restorativeness directly, capturing a true and meaningful direct pathway requires accounting for unobserved confounding variables and participant heterogeneity. Because our current model relies on a composite index that merges duration and intensity, it lacks the granularity to isolate how individual differences in specific exercise modalities (14) or contextual influences such as social interaction status (e.g., exercising alone versus with companions) (15) differentially shape cognitive demands and environmental engagement, thereby obscuring the true direct relationship.

In contrast, the interaction effect of exercise behavior and perceived naturalness on restorativeness (*β* = -.081, *f^2^* = .009), though low by conventional standards, holds substantive practical value. According to the criteria for moderation effects in applied social sciences, any interaction effect size exceeding.005 is highly meaningful and worthy of empirical discussion (30). This negative interaction empirically demonstrates that exercise behavior functions as a critical boundary condition that attenuates the positive relationship between environmental naturalness and restorativeness. A plausible explanation, through the lens of cognitive resource allocation, is that higher exercise workloads may trigger a potential cognitive resource competition, suggesting that intense physical strain prompts individuals to shift their directed attention inwardly toward physiological signals and fatigue tolerance. Although this real-time cognitive mechanism was not directly measured in our survey, this inward attentional shift offers a strong theoretical interpretation for why increased physical exertion appears to interfere with the cognitive processing of external restorative settings.

This interpretation directly corroborates the findings of Barton and Pretty (32), who maintained that psychological benefits often peak during short, light-intensity engagements and exhibit diminishing returns as exercise intensity and duration increase. This finding further supports the argument that high physical effort shifts attention internally, aligning with recent green exercise research that emphasizes the critical role of dynamic human-environment interactions and an individual’s limited capacity to actively engage with restorative natural surroundings under physical strain (33). Ultimately, this confirms the complex relationship between physical activity and the health benefits derived from green spaces, highlighting that vigorous or prolonged exercise can indeed attenuate the intrinsic restorative premium provided by nature.

### 4.3 Theoretical implications

These results provide crucial insights into the psychological mechanisms underlying green exercise, offering two key theoretical contributions. First, by confirming nature relatedness as a critical mediator, this study empirically validates CRT (6). This finding further supports the argument that the theoretical paradigm must shift from the purely environment-centric perspectives of SRT and ART to a person-environment interaction model. It demonstrates that the psychological benefits of green exercise depend heavily on activating an individual’s implicit connection with nature.

Second, integrating a cognitive resource allocation perspective challenges the pervasive assumption that combining nature and exercise universally yields additive benefits. Our results suggest that high-intensity green exercise induces a dual-task cognitive interference. Specifically, vigorous exertion forces an inward shift in directed attention, depleting the cognitive resources required to process external restorative environments. This perspective bridges exercise physiology and environmental psychology, effectively explaining the attenuating interaction effect observed at higher physical workloads.

### 4.4 Practical implications

An implication of these findings is the potential for optimizing both environmental design and public health guidelines. For urban planners and landscape architects, simply increasing green coverage is insufficient; design strategies must actively foster human-nature interactions. Incorporating elements such as sensory gardens or accessible ecological trails can cultivate NR and maximize the restorative potential of urban spaces. For public health professionals and sports coaches, exercise intensity must be carefully calibrated against the environmental context. To optimize psychological restoration, a “moderate dose” approach (e.g., brisk walking or light jogging) is strongly recommended. Mitigating the cognitive depletion caused by vigorous workouts enables individuals to fully immerse themselves in nature, thereby unlocking the true synergistic mental health benefits of green exercise.

### 4.5 Limitations and future research

While this study offers significant theoretical and practical contributions, it is subject to several limitations that suggest directions for future research. As highlighted in the discussion of the interaction effect, the operationalization of exercise behavior did not differentiate among specific exercise modalities (14) or account for social interaction status (e.g., green exercise alone versus in groups) (15). These unmeasured factors introduce varying cognitive demands and levels of environmental engagement, potentially obscuring both the direct path and the absolute magnitude of the interaction. Future studies should incorporate diverse exercise typologies and social contexts to refine how these variables dynamically moderate restorative pathways.

Additionally, PN was operationalized as a single continuum from primitive nature to urban development based on the ROS framework. Although this single-item measure effectively reduced respondent burden, PN is a complex construct involving multiple dimensions, such as biodiversity and vegetation density. This psychometric reduction may compromise construct validity and attenuate path coefficients; thus, our model’s findings should be interpreted as a conservative estimate. Future research should employ multidimensional scales to capture these complex environmental attributes.

Although based on a robust, large-scale sample, the findings are inherently constrained by potential cultural biases, as green exercise experiences are profoundly shaped by sociocultural contexts (34). For instance, certain philosophical traditions interpret NR as an introspective, tranquil, and harmonious state of mind-body integration (35). Moreover, individuals embedded in these cultural contexts may enter natural environments with strong positive anticipation, establishing pre-existing mental conditioning that potentially magnifies the psychological benefits of green exercise (36). Consequently, these cultural and cognitive nuances imply that the dynamic conditioned activation pathways of CRT might fluctuate significantly across diverse populations. Future inquiries therefore require cross-cultural validation to confirm the universal applicability of both CRT and the cognitive resource competition framework across diverse geographical and cultural settings.

## Data Availability

The datasets presented in this article are not readily available because of the privacy regulations and ethical guidelines aimed at protecting participant confidentiality. Specifically, public sharing of the data was not included in the approved ethical protocol by the Research Ethics Committee, National Taiwan Normal University. Providing unrestricted public access could potentially compromise the privacy of the participants. Requests to access the datasets should be directed to T-CH, 81031008a@gapps.ntnu.edu.tw.
