# Supplementary material for: The psychological restorative effects of green exercise: a moderated mediation model of nature relatedness and exercise behavior
Source: Front Sports Act Living. 2026 Jul 9;8:1887724. doi: 10.3389/fspor.2026.1887724 (PMC13391835; doi:10.3389/fspor.2026.1887724)
Supplement: Supplementary file 1 [file Supplementaryfile1.docx]

Supplementary Material

# Supplementary Table

| **Supplementary Table 1.** PLS-SEM Model Evaluation | | | | | | |
| --- | --- | --- | --- | --- | --- | --- |
| Pathway | *β* | Mean | SD | *t* | *f^2^* | Effect Size |
| *Direct effect* | | | | | | |
| PN → PER | .103 | .103 | .024 | 4.313^***^ | .015 | No effect |
| PN → NR | .152 | .152 | .029 | 5.170^***^ | .024 | Medium effect |
| NR → PER | .536 | .538 | .027 | 20.038^***^ | .415 | Large effect |
| EB → PER | .063 | .065 | .028 | 2.242^*^ | .006 | No effect |
| *Moderation* | | | | | | |
| EB x PN →PER | -.081 | -.075 | .028 | 2.913^**^ | .009 | Small effect |
| *Mediation* | | | | | | |
| PN → NR → PER | .081 | .081 | .016 | 5.200^***^ | -- | -- |
| **p < .05, **p < .01, *** p < .001* | | | | | | |

**Supplementary Table 2.** Information of the Expert Panel for Questionnaire Development

| **Code** | **Affiliation** | **Expertise** |
| --- | --- | --- |
| S1 | National Tsing Hua University | Active Aging Sports Instruction, Intergenerational Learning |
| S2 | National Chengchi University | Sport Organizational Behavior, Sport Policy |
| S3 | National Taiwan University | Exercise Physiology, Sports Training |
| S4 | University of Taipei | Sport Facility Operations and Management |
| S5 | National Taiwan Sport University | Sport Policy, Physical Education Administration and Management |
| S6 | XPORTS SPORTS CO., LTD. | Sports Marketing, Sport Facility Management |

**Supplementary Table 3.** Full Collinearity VIF Results for CMV Assessment

| **Constructs** | **Inner VIF (to Dummy)** | **Conclusion** |
| --- | --- | --- |
| EB | 1.053 | Pass (< 3.3) |
| NR | 1.441 | Pass (< 3.3) |
| PER | 1.446 | Pass (< 3.3) |
| PN | 1.081 | Pass (< 3.3) |
